# The Listening Network and Cochlear Implant Benefits in Hearing-Impaired Adults

**DOI:** 10.3389/fnagi.2021.589296

**Published:** 2021-02-25

**Authors:** Chris J. James, Petra L. Graham, Frank A. Betances Reinoso, Silvia N. Breuning, Marcin Durko, Alicia Huarte Irujo, Juan Royo López, Lida Müller, Adam Perenyi, Rafael Jaramillo Saffon, Sandra Salinas Garcia, Mark Schüssler, Margarita J. Schwarz Langer, Piotr H. Skarzynski, Dianne J. Mecklenburg

**Affiliations:** ^1^Cochlear France SAS, Toulouse, France; ^2^Department of Mathematics and Statistics, Macquarie University, North Ryde, NSW, Australia; ^3^Servicio de Otorrinolaringología, Hospital Universitario Donostia, Madrid, Spain; ^4^Centro de Investigaciones Otoaudiológicas, Buenos Aires, Argentina; ^5^Department of Otolaryngology, Head and Neck Oncology, Medical University of Lodz, Lodz, Poland; ^6^Department of Otorhinolaryngology, Clínica Universidad de Navarra, Pamplona, Spain; ^7^Servicio de Otorrinolaringología, Hospital Clínico Universitario Lozano Blesa, Zaragoza, Spain; ^8^Tygerberg Hospital—Stellenbosch University Cochlear Implant Unit, Tygerberg, South Africa; ^9^Department of Otolaryngology and Head Neck Surgery, Albert Szent Györgyi Medical Center, University of Szeged, Szeged, Hungary; ^10^Consultorio Jaramillo, Manizales, Columbia; ^11^Servicio de Otorrinolaringología y Patología Cérvico-Facial, Fundación Jiménez Díaz University Hospital, Madrid, Spain; ^12^Deutsches HörZentrum Hannover der HNO-Klinik, Medizische Hochschule Hannover, Hannover, Germany; ^13^Clinica Orlant, Medellin, Colombia; ^14^Institute of Physiology and Pathology of Hearing, Warsaw, Poland; ^15^Cavale International, Basel, Switzerland

**Keywords:** hearing loss, cochlear implant, speech spatial and qualities of hearing scale, age effect, hemispheric dominance, quality of life

## Abstract

Older adults with mild or no hearing loss make more errors and expend more effort listening to speech. Cochlear implants (CI) restore hearing to deaf patients but with limited fidelity. We hypothesized that patient-reported hearing and health-related quality of life in CI patients may similarly vary according to age. Speech Spatial Qualities (SSQ) of hearing scale and Health Utilities Index Mark III (HUI) questionnaires were administered to 543 unilaterally implanted adults across Europe, South Africa, and South America. Data were acquired before surgery and at 1, 2, and 3 years post-surgery. Data were analyzed using linear mixed models with visit, age group (18–34, 35–44, 45–54, 55–64, and 65+), and side of implant as main factors and adjusted for other covariates. Tinnitus and dizziness prevalence did not vary with age, but older groups had more preoperative hearing. Preoperatively and postoperatively, SSQ scores were significantly higher (Δ0.75–0.82) for those aged <45 compared with those 55+. However, gains in SSQ scores were equivalent across age groups, although postoperative SSQ scores were higher in right-ear implanted subjects. All age groups benefited equally in terms of HUI gain (0.18), with no decrease in scores with age. Overall, younger adults appeared to cope better with a degraded hearing before and after CI, leading to better subjective hearing performance.

## Introduction

Cochlear implants (CI) are the treatment of choice to restore the reception of sound when hearing aids no longer provide an adequate level of speech understanding for the listener to function in everyday situations. A CI bypasses the peripheral system, replacing acoustic input with electrical signals and thus reestablishing the means to receive sensory information and activate neural transmission, albeit with considerably reduced fidelity compared with normal hearing (Olds et al., 2016; Dorman et al., 2017).

A significant level of background noise requires listeners with intact hearing to exert more effort to receive and repair target speech, such as by utilizing strategies of auditory closure (Madix, 2006). However, even in noise-free situations, CI users and other hearing-impaired individuals need to expend significant cognitive resources to hear and understand the message (Stahl, 2017). Also, older adults even with mild or no hearing loss make more errors and expend more effort listening to speech (Roberts and Allen, 2016; Degeest et al., 2017; Peelle, 2018). Hearing is receiving auditory signals, and listening is cognitively organizing what has been received. The CI takes advantage of neural reserve pathways that transmit the information to the cortex for interpretation. A CI reactivates broader stimulation within the auditory periphery (Amichetti et al., 2018; Sardone et al., 2019), but can only convey the sound in a limited fashion, with potential bottlenecks still arising in low-level retrocochlear processes. By contrast, listening is a top-down process (André et al., 2019) and may, therefore, be impacted by the capacity of listening processes further up the network that relies on cognitive function and may suffer age-related impairment (Rosemann and Thiel, 2019).

Listening takes place when the receiver actively intends to understand. However, defining listening as a process is too narrow because it cannot be separated from the anatomical, physiological and psychosocial circumstances that impact it. A “listening network” was first coined in a study of complex music structures (Meeùs, 1993); however, we propose a more comprehensive concept of a listening network that, in the case of speech and language communication, is driven by functional access to auditory pathways (bottom-up processing) and cortical function (top-down processing), can also be considered to serve as a link between the two and adapts to environmental influences by repurposing, as needed (Amichetti et al., 2018). Peelle and Wingfield (2016) refer to an extended cortical network but the listening network considers all aspects involved in receiving, transmitting, and comprehending meaningful sound information, including a deafened adult’s condition or use of a prosthesis, such as a CI, that potentially causes perceptual distortions.

The principal function of cochlear implantation in adults is to restore receptive speech recognition (James et al., 2019). Language appears to be specialized to the left hemisphere, whether it is spoken language or visually perceived language such as lipreading and sign language (Scott and McGettigan, 2013). It has thus long been posited that unilateral cochlear implantation (as in this cohort) may be more impactful in the right ear due to contralateral sensory brain organization. Neurophysiological preference at this level may have a positive effect on the listening network and, therefore, outcomes.

Considering negative impacts to the listening network: at the level of reception, it encompasses all types of peripheral hearing loss and conditions that limit or degrade input signals (degraded acoustic information, poorly articulated speech, et cetera); at the level of transmission, all forms of retrocochlear pathology (auditory neuropathies, synaptopathy, see also Shearer and Hansen, 2019), and; at the level of comprehension, by cortical dysfunction linked to emotional and environmental factors. Any interference within the network will increase listening effort, decrease speech understanding, and ultimately decrease quality of life (QoL) and, conversely, any influence that enhances network function will reduce listening effort, improve performance and potentially increase the quality of life.

An inevitable disruption to the listening network is age-related hearing loss (ARHL), which is a pervasive, progressive disorder commonly referred to as presbycusis (Sardone et al., 2019). Contemporary science describes ARHL, aside from its genetic component, as a synaptopathy in which intact hair cells lose their synaptic properties thus reducing afferent neural efficiency (Liberman et al., 2016; Tu and Friedman, 2018). This has the effect of reducing the quality of input signals rather than completely depriving the brain of sound. Although mainly associated with affecting the transmission of the temporal characteristics of auditory signals (Sergeyenko et al., 2013; Parthasarathy and Kujawa, 2018; Profant et al., 2019), distortion caused by ARHL occurs at all levels of the auditory system (Ren et al., 2013; Profant et al., 2019).

When the cortical regions responsible for interpreting auditory information receive poor or limited signals, the brain recruits other associated regions (Fortunato et al., 2016; Peelle and Wingfield, 2016; Lazard and Giraud, 2017; Eggermont, 2019) to aid in understanding and to manage the anticipation of a task perceived to be effortful (Vassena et al., 2019; Müesch et al., 2020). This can have several repercussions such as cortical overload, functional reorganization, increased listening effort, and cognitive decline (Erb and Obleser, 2013; Ayasse et al., 2017, and see reviews by Cardin, 2016 and Amieva and Ouvrard, 2020).

ARHL may further reduce speech understanding performance when coupled with peripheral sensorineural hearing loss (Humes et al., 2013). Most recently, Hey et al. (2020) reported differences of as much as 30% in word recognition scores in quiet between older and younger CI users, with differences attributed to age and not level of hearing loss. Kim et al. (2018) demonstrated the mismatches due to age between word scores and level of hearing loss—both in modeling studies and in human cases.

Despite such mismatches, it remains unclear which factors most affect benefit for those receiving a CI in adulthood. Self-report questionnaires have typically been employed to probe how listeners perceive performance changes after the restoration of sound sensation (Vannson et al., 2015; Sarant et al., 2020; Warringa et al., 2020). Two widely applied scales are the Speech, Spatial, and Qualities (SSQ) of Hearing scale (Gatehouse and Noble, 2004) to evaluate everyday hearing performance, and the Health Utilities Index Mark III (HUI; Feeny et al., 2002) to investigate overall changes in health-related quality of life. The SSQ is a validated hearing-specific scale developed to aid in the evaluation of the subjective aspects of listening experience encountered in a variety of everyday situations and, reportedly, is the only extensively used questionnaire that addresses listening effort (Alhanbali et al., 2017). The HUI is a general health utility-scale that includes a wide range of attributes of which hearing is one of eight (vision, hearing, speech, ambulation, dexterity, emotion, cognition, and pain) yielding a value for overall health status and each specific attribute (Noto and Uemura, 2020). However, given the generic status of the different attributes, it is therefore likely that other age-related changes to the body may also affect outcomes on this scale.

The availability of self-reported listening performance and health-related quality of life in a large cohort of more than 500 adult first-time users of a unilateral CI presented an opportunity: within the framework of the listening network, we wanted to explore the effect of implant side and age on self-reported hearing performance with the SSQ, and quality of life employing the HUI.

## Materials and Methods

### Data Source and Subjects

Data from subjects who received Nucleus^®^ cochlear implants (Cochlear Limited, Macquarie University, NSW, Australia) were extracted from the Implant Recipient Observational Study (IROS) database (data accessed March 25, 2020) described by Lenarz et al. (2017). Data were collected from 12 implant centers across seven countries in Europe, South Africa, and South America. These centers were chosen based on the completeness of data records. Languages spoken by subjects were predominantly Afrikaans, English, German, Hungarian, Polish and Spanish. Selection criteria were age >18 at time of implantation, unilateral cochlear implantation, and no second implant during the period of follow-up (maximum 3 years). Included patients were implanted between 2011 and 2019, and thus used Nucleus CP810 or CP900 series sound processors. Approximately 60% were implanted with Contour Advance and 15% Slim Modiolar perimodiolar-type electrode arrays, and the remaining 25% “Slim” straight electrode arrays. No radiographical data were available to indicate array position in the cochlea or the presence of dislocations out of scala tympani.

### Materials

Standard audiometric pure-tone threshold data were collected for each subject at baseline (pre-implant) for frequencies 500, 1,000, 2,000, and 4,000 Hz. These values were categorized into standard degrees of hearing loss (HL) which represent ranges of four-frequency pure-tone average (PTA) thresholds as mild 26–40 dB HL, moderate 41–55 dB HL, moderately severe 56–70 dB HL, severe 71–90 dB and profound >90 dB HL.

The Speech, Spatial, and Qualities of Hearing Scale (SSQ) is a validated, disease-specific, self-report questionnaire specifically designed for hearing-impaired individuals (Gatehouse and Noble, 2004). It consists of 49 questions that allow an individual to rate the perceived disability in different communication circumstances on a visual analog scale of 0–10. Results are summarized as a total score, as reported here, or reported by the subcategories of speech understanding, spatial perception, and quality of speech sounds. Study participants were directed to complete the questionnaire on their own.

The Health Utilities Index Mark 3 (HUI) is one of the most widely applied self-report rating scales in studies describing the health-related quality of life in hearing-impaired adults. As a multi-attribute health scale, it covers vision, hearing, speech, ambulation, dexterity, emotion, cognition, and pain. Study participants were directed to complete the HUI questionnaire, consisting of 17 questions on their own. HUI Mark 3 scores range from zero (dead) to one (perfect health), but can also be less than zero in the case of depression combined with other low scores (worse than dead).

Demographics, comorbidities, and limited hearing history, and audiometric data were also collected. Here, we considered the ear of implant; the degree of HL; presence or absence of tinnitus and dizziness; hearing aid and telephone use; and duration of HL.

### Statistical Considerations

Kruskal–Wallis one-way analysis of variance (ANOVA) and chi-squared tests were used to determine whether the continuous and categorical predictors, respectively, differed between age groups.

Linear mixed-effects models were developed to determine the effects of age group and side of implant (left, right) on SSQ and HUI scores. An interaction between age group and visit or implant side and visit were included in the model to determine whether change over time was different for specific age groups or implant sides. In the linear mixed-effects models, a random intercept for each person was used to account for the correlation induced by repeated measurements. Linear mixed-effects models use all available data to give an unbiased estimate of mean effect assuming data lost to follow-up are missing at random (Ibrahim and Molenberghs, 2009).

All linear mixed-effects models were adjusted for factors potentially associated with a change in SSQ and HUI scores. These factors included gender, baseline presence of tinnitus, degree of HL in the contralateral ear, duration of deafness, and use of hearing aids at baseline. Tukey pairwise tests were used to determine which pairs of age groups differed and the differences between the side of implantation in linear mixed-effects models. *P*-values less than 0.05 were considered significant.

## Results

### Age Grouping

The numbers of subjects for whom baseline and 1-, 2- and 3-year follow-up data points were available are given in Table 1. Age grouping at baseline was defined in decade ranges except that those aged 65+ were combined into one group, as were those aged <35, to avoid small group sizes at baseline and over time. There were no systematic differences between age groups in the type of devices used.

**Table 1:** Numbers of records in the implant recipient observational study (IROS) database for unilateral cochlear implants (CI) subjects meeting the inclusion criteria.

	Number (%) of subjects by age range at each visit					
Interval	All	[18–34]	[35–44]	[45–54]	[55–64]	[65–93]
Baseline	543	81 (15%)	80 (15%)	94 (17%)	120 (22%)	168 (31%)
1-year	416	66 (16%)	61 (15%)	71 (17%)	93 (22%)	125 (30%)
2-year	305	49 (16%)	49 (15%)	50 (17%)	69 (23%)	88 (29%)
3-year	154	28 (18%)	25 (16%)	26 (17%)	38 (25%)	37 (24%)

The proportion of younger subjects increased with the follow-up period while the proportion of oldest subjects decreased. There were no significant group differences in the baseline characteristics of those who were followed up to 1 year and those who were not.

### Baseline Characteristics

The etiology of hearing loss is given in Table 2. The largest group of cases (43%) had no identified cause of hearing loss. It should be noted that sudden deafness and family-linked deafness often have no localizable lesion to explain the hearing loss and thus are poorly defined. Otosclerosis, ototoxic drugs, and trauma had well-defined effects on peripheral auditory physiology but together only accounted for 15% of subjects.

**Table 2:** Etiology of hearing loss.

Etiology	*N*	(%)
Acoustic neuroma	5	(0.9)
Bacterial infection	9	(1.7)
Cholesteatoma	5	(0.9)
Chronic otitis	14	(2.6)
Congenital atresia	4	(0.7)
Familial	33	(6.1)
Large vestibular aqueduct	3	(0.5)
Measles	5	(0.9)
Meniere’s syndrome	21	(3.9)
Meningitis	16	(2.9)
Mondini syndrome	1	(0.2)
Mumps	3	(0.5)
Noise exposure	8	(1.5)
Otosclerosis	34	(6.3)
Ototoxic drugs	25	(4.6)
Rubella	6	(1.1)
Sudden deafness	44	(8.1)
Trauma	15	(2.8)
Viral infection	4	(0.7)
Unknown	232	(42.6)
Other	56	(10.3)

Table 3 summarizes the baseline characteristics by age group. At baseline, 56% of subjects were female and there were significantly more women in the younger age groups. More than half of the subjects were implanted in the right ear (313/543 or 58%) and significantly more right-hand-side implantees were in the younger age groups. Only 42/543 (8%) subjects had an additional handicap: 27 had syndromic or other multiple handicaps, 11 had impaired mobility and three were blind.

**Table 3:** Number (%) of subjects or median (IQR) for each of the baseline variables by age grouping.

	All	18–34	35–44	45–54	55–64	65–93	*P*-value
	(*n* = 543)	(*n* = 81)	(*n* = 80)	(*n* = 94)	(*n* = 120)	(*n* = 168)
Female	304 (56.0)	53 (65.4)	50 (62.5)	59 (62.8)	54 (45.0)	88 (52.4)	0.012
Right hand side implant	313 (57.6)	59 (72.8)	52 (65.0)	54 (57.4)	54 (45.0)	94 (56.0)	0.002
Additional handicap	42 (7.8)	5 (6.2)	6 (7.6)	3 (3.2)	11 (9.2)	17 (10.3)	0.302
HL degree (implant ear)							0.163
Mild-moderately severe	26 (5.0)	2 (2.5)	2 (2.7)	2 (2.3)	8 (6.8)	12 (7.5)	
Severe	117 (22.5)	18 (22.5)	11 (14.7)	19 (22.1)	26 (22.2)	43 (27.0)	
Profound	374 (72.3)	60 (75.0)	62 (82.7)	65 (75.6)	83 (70.9)	104 (65.4)	
HL degree (non-implant ear)							0.032*
Normal	28 (5.5)	4 (5.1)	4 (5.4)	8 (9.3)	8 (6.9)	4 (2.6)	
Mild-moderately severe	82 (16.1)	7 (9.0)	7 (9.5)	12 (14.0)	22 (19.0)	34 (21.8)	
Severe	133 (26.1)	22 (28.2)	14 (18.9)	29 (33.7)	27 (23.3)	41 (26.3)	
Profound	267 (52.4)	45 (57.7)	49 (66.2)	37 (43.0)	59 (50.9)	77 (49.4)	
Presence of tinnitus	283 (53.2)	33 (42.9)	46 (59.0)	58 (62.4)	62 (51.7)	84 (51.2)	0.127
Presence of dizziness	238 (44.7)	27 (35.1)	29 (37.2)	48 (51.6)	55 (45.8)	79 (48.2)	0.119
Hearing aid (implant ear)	329 (60.6)	49 (60.5)	50 (62.5)	53 (56.4)	71 (59.2)	106 (63.1)	0.851
Hearing aid (non-implant ear)	338 (62.2)	46 (56.8)	45 (56.2)	65 (69.1)	72 (60.0)	110 (65.5)	0.271
Telephone use	281 (52.8)	43 (55.8)	39 (50.0)	55 (59.1)	68 (56.7)	76 (46.3)	0.240
Median duration of HL (years)	21 (25)	21 (15)	28 (31)	20 (26)	17 (34.5)	20 (19)	0.661

P-values are from chi-squared tests for categorical variables and analysis of variance for continuous variables; HL, hearing loss; IQR, interquartile range; *Fisher’s exact test used because of small counts.

There were no significant differences in the proportions of subjects using hearing aids or telephone, or the presence of tinnitus between age groups at baseline (Table 3). The presence of dizziness was similar across age groups at baseline. Prevalence of continuous dizziness was 7–10% at 1 year for 45+ age groups, but <2% for younger age groups.

Overall, about 95% of subjects were severely or profoundly deaf in the implanted ear (Table 3, hearing loss degree), and about 78% in the non-implant ear (right panel). The degree of hearing loss did not significantly vary with the age group for the implanted ear but did for the non-implant ear (Table 3): there were higher proportions of severe or profound hearing loss in non-implant ears of younger subjects compared with older subjects.

The median duration of hearing loss was greatest at 28 years in the 35–44 age group and least at 17 years in the 55–64 age group. However, there was no statistically significant difference in duration of hearing loss by age group.

### Age Group Differences in Primary Outcome Measures

**Figure 1:**
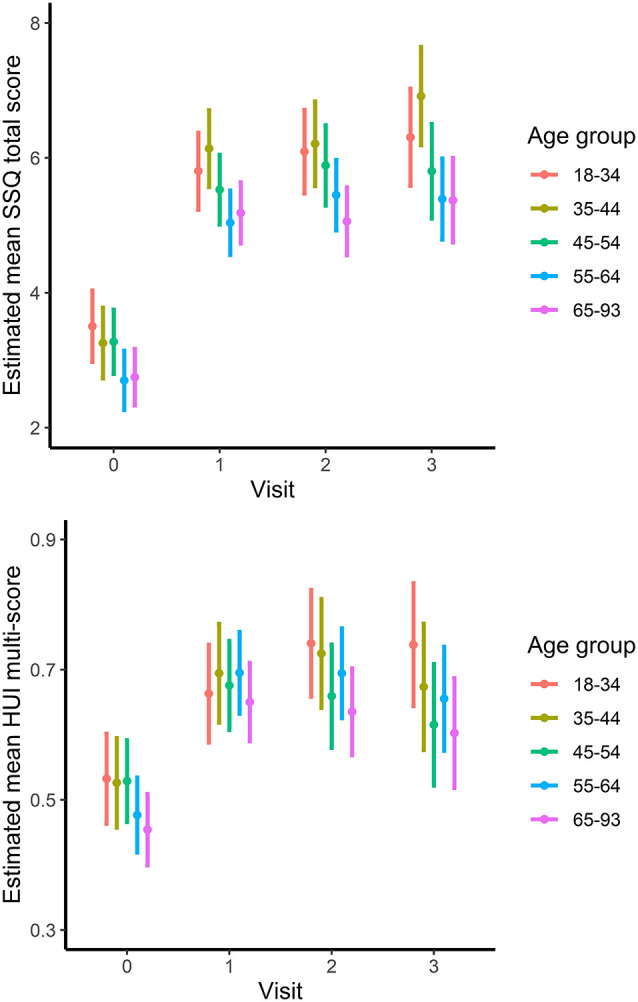
SSQ (upper) and HUI (lower) scores by visit for each age group. Points and lines indicate least-squares means with 95% confidence intervals.

Analyses of the changes in SSQ and HUI scores over time and between age groups are presented in Table 4 and Figure 1. Results show that there was no significant interaction between visit and age group for either SSQ or HUI scores, implying that the age groups performed similarly over time. The main effect for visits was significant (*p* < 0.001) suggesting that SSQ scores improved over time (Figure 1). In fact, SSQ scores increased significantly between baseline and 1-year by 2.4 units, further increasing by 0.39 points between 1 year and 3 years. The main effect for age group was also significant; SSQ scores differed between age groups and differences were constant over time. There was a significant change over time in HUI scores but no other significant effects.

**Table 4:** Summary statistics for the linear mixed-effects model for speech spatial qualities (SSQ) and health utilities index mark III (HUI) scores.

	Mean squares	Numerator DF	Denominator DF	*F*	*P* (>*F*)
**SSQ**					
**Visit**	533.3	3	800.9	301.9	**<0.001**
**Age group**	13.79	4	504.5	7.808	**<0.001**
Visit × Age group	1.82	12	804.0	1.032	0.417
*Implant side*	6.27	1	445.1	3.547	0.060
*Gender*	0.31	1	449.2	0.174	0.677
*Presence of tinnitus*	4.05	1	439.0	2.294	0.131
*Duration of deafness*	0.02	1	440.7	0.009	0.924
**Hearing aid use**	43.26	1	447.6	24.49	**<0.001**
**HL degree (non-implant ear)**	28.81	5	491.3	16.31	**<0.001**
**HUI**					
**Visit**	2.467	3	800.7	77.16	**<0.001**
**Age group**	0.076	4	507.7	2.391	**0.050**
Visit × Age group	0.036	12	804.0	1.115	0.344
*Implant side*	0.011	1	445.1	0.346	0.557
*Gender*	0.115	1	448.5	3.602	0.058
*Presence of tinnitus*	0.071	1	436.9	2.236	0.136
*Duration of deafness*	0.008	1	439.3	0.266	0.606
**Hearing aid use**	0.233	1	449.9	7.284	**0.007**
**HL degree (non-implant ear)**	0.248	5	492.3	7.754	**<0.001**

*Main effects of visit and age group plus their interaction were modelled with adjustment for other covariates. Significant results are in bold*.

The effect of age group on SSQ scores is represented in Figure 1 (top). Those aged <45 had significantly higher scores than those 55 or older and the difference was constant over time, with scores for the 45–54 age group falling between the two. Tukey pairwise tests indicated that the 18–34- and 35–44-year-old age groups had significantly higher SSQ scores than the 55–64 age group (*p* = 0.002 and *p* = 0.009, respectively) and the 65–93 age group (*p* < 0.001 and *p* = 0.005). Differences were 0.82 points between the youngest age group and those in age groups 55–93, and 0.75 points between the 35–44-year olds and those in age groups 55–93. There was approximately zero difference (0.003, *p* = 1.000) in SSQ scores between those in the 55–64 and 65–93-year-old groups.

Separate analyses of the SSQ subscale scores, speech recognition, spatial awareness, and sound quality, mirrored the pattern for SSQ total scores (not presented).

Mean improvement in HUI score, displayed in Figure 1 (bottom), was 0.18 units from baseline to visit 1 (*p* < 0.001) with a slightly smaller estimated increase from baseline to visit 3 (0.015, *p* < 0.001), but there was no statistically significant difference between age groups or interaction effect (Table 4). A separate analysis of the HUI hearing attribute scores alone also indicated no age-group difference.

### Side of Implant Differences for Primary Outcome Measures

SSQ and HUI scores were further compared in a linear mixed-effects model for the interaction between implant side and visit based on the models given in Table 5. Overall outcomes remained the same as in Table 4 for the main effect of age group.

**Table 5:** Summary statistics for the linear mixed-effects model for SSQ and HUI scores.

	Mean squares	Numerator DF	Denominator DF	*F*	*P* (>*F*)
**SSQ**					
**Visit**	522.5	3	814.9	298.3	**<0.001**
**Age group**	11.91	4	436.0	6.800	**<0.001**
**Implant side**	11.92	1	526.4	6.808	**0.009**
**Implant side × visit**	5.80	3	816.8	3.311	**0.020**
*Gender*	0.41	1	448.6	0.231	0.631
*Presence of tinnitus*	3.75	1	439.5	2.139	0.144
*Duration of deafness*	0.01	1	440.8	0.003	0.954
**Hearing aid use**	43.63	1	446.9	24.91	**<0.001**
**HL degree (non-implant ear)**	29.29	5	491.2	16.72	**<0.001**
**HUI**					
**Visit**	2.774	3	818.0	86.57	**<0.001**
Age group	0.074	4	436.3	2.309	0.057
Implant side	0.009	1	532.3	0.295	0.587
Implant side × visit	0.103	3	819.9	1.069	0.361
*Gender*	0.121	1	449.4	3.771	0.053
*Presence of tinnitus*	0.069	1	438.7	2.165	0.142
*Duration of deafness*	0.009	1	440.5	0.279	0.597
**Hearing aid use**	0.249	1	450.4	7.767	**0.006**
**HL degree (non-implant ear)**	0.246	5	494.2	7.671	**<0.001**

*The main effects of visit and implant side plus their interaction were modelled with adjustment for other covariates. Significant results are in bold*.

**Figure 2:**
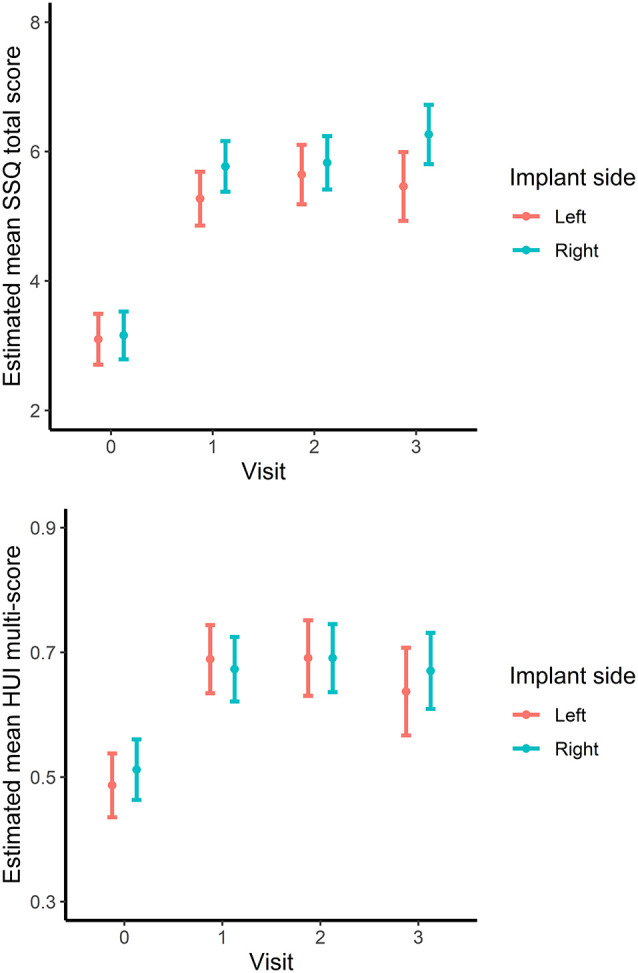
SSQ (upper) and HUI (lower) scores by visit for left- and right-ear implanted subjects. Points and lines indicate least-squares means with 95% confidence intervals.

There was a significant effect of implant side and interaction between visit and implant side for SSQ scores (Table 1) indicating some differences between left and right ears across visits. This is illustrated in Figure 2 (top). The difference between the left and right sides was only 0.07 SSQ units (*p* = 0.670) at baseline. However, SSQ scores were significantly greater for right-implanted subjects than for left-implanted subjects at 1 year (0.49 units, *p* = 0.008) and 3 years (0.79 units, *p* = 0.005) but not at 2 years (0.15 units, *p* = 0.511). The difference in mean SSQ scores for the side of the implant was slightly greater than half that of the age-group effect.

For HUI scores, there was no significant interaction between visit and implant side (*p* = 0.323) and no main effect of implant side (*p* = 0.639) indicating no evidence of an association between implant side and change in HUI scores (Figure 2, bottom).

### Secondary Measures of Outcomes Related to Hearing

Tinnitus states changed significantly from baseline to year 1 (McNemar’s, $\chi_{\left(\text{3}\right)}^{\text{2}}$ = 11.8, *p* < 0.01) with overall improvements in the rate of reported tinnitus; for example, 50% reported tinnitus at 1 year compared with 58% at baseline. Tinnitus state changed in both directions such that 60 of the 201 with tinnitus at baseline reported no tinnitus at 1 year, but 34 of 145 with no tinnitus at baseline reported tinnitus at 1 year. Dizziness states also changed significantly (McNemar’s tests, $\chi_{\left(\text{10}\right)}^{\text{2}}$ = 41, *p* < 0.001) where more than 50% of subjects reported a less severe state and <10% a more severe state.

Telephone use improved significantly from baseline to 1 year (McNemar’s tests, $\chi_{\left(\text{3}\right)}^{\text{2}}$ = 90, *p* < 0.001): 70% of those who did not use the telephone at baseline reported using the telephone at 1 year. Conversely, only 10% of those who did use the telephone at baseline stopped using it at 1 year. Telephone use did not vary significantly by age group either at baseline or 1 year after CI.

## Discussion

### Main Findings

Our study examined the relationship between the side of implantation and age and self-reported hearing performance and health status in 543 hearing-impaired adults receiving a cochlear implant in one ear. To this end, we divided subjects into approximate decade age groups, with wider ranges for the youngest (18–35 years) and oldest (65–93 years) groups to allow for sufficiently high numbers for analysis. As shown in Figure 1 by SSQ scores, hearing performance differed significantly between age groups both at baseline and after 1–3 years of use of the CI. Specifically, subjects in age groups <45 reported greater SSQ scores than those in age groups 55+. Also, SSQ scores post-implantation were significantly higher for right-ear implanted subjects. By adjusting the models for baseline covariates, such as degree of HL in the non-implant ear (by far most often the ear with more residual hearing), hearing aid use, and side of implantation, we can be confident that this was not due to the differences in hearing characteristics between age groups. Younger subjects tended to have more bilateral hearing loss compared with older subjects, though not significantly more in their implanted ear (Table 3). A similar situation was observed in a recent study by Hey et al. (2020) where candidacy criteria for CI are generally based on speech recognition scores obtained in laboratory conditions. In their study, speech recognition scores were on average lower for older subjects despite lower degrees of hearing loss, and this continued postoperatively with the use of CI. If the capacity to manage sensorineural hearing loss decreases with age, then CI candidates will tend to have lower degrees of peripheral hearing loss with increasing age. Scores on all SSQ subscales varied with age. At least one study has also noted that spatial hearing, not just speech perception, is affected both by age and peripheral HL (Gallun et al., 2013).

Self-reported health utility measured by HUI scores did not significantly vary across age groups, except for those aged >70. It is expected that older CI-users would report lower scores given the multi-dimensional weighting where just one other health problem may have an impact (see Grutters et al., 2007; Feeny et al., 2018). But, as for SSQ, there was a significant gain in HUI scores between baseline and post-implant, with the largest change after 1 year of experience with the CI.

### Functional Benefits of Cochlear Implantation

Pre-implant use of hearing aids as well as ongoing use of telephone may contribute to receiving useful auditory information that helps to maintain more-normal functioning of the listening network (Wongrakpanich et al., 2016; Ohlenforst et al., 2017; Sarant et al., 2019). Use of hearing aids and telephone use did not vary with age group at baseline. Unlike hearing aid use, telephone use significantly increased after 1–2 years’ experience. The willingness to attempt telephone communication increased over-time with CI experience and may be relevant to post-implant gains in benefit. Similarly, proactive training can also enhance or maintain performance and normal processing (e.g., Anderson et al., 2013).

Tinnitus and dizziness are related to hearing health and negatively affect the quality of life (Grauvogel et al., 2010; Miura et al., 2017; Chang et al., 2019). We did not observe a prevalence difference across age groups for tinnitus as has been reported by other authors (Sindhusake et al., 2003; McCormack et al., 2016) or was there an indication that tinnitus increases with age as suggested by Al-Swiahb and Park (2016) and reviewed by McCormack et al. (2016). It remains unclear whether age is a primary factor in predicting the presence of tinnitus; for instance, whether earlier exposure to noise-induced HL may be a greater influence than progressive effects of ARHL (Krug et al., 2015). Although both dizziness and tinnitus are more often reported by those with ARHL, no age effect was observed for either in this study. Epidemiological studies on tinnitus reveal challenges in valid estimations due to variations in definitions of tinnitus, small group sizes studied, and differences in evaluation methods (McCormack et al., 2016); for instance, in small sample size, an even greater incidence of 79% was described for a group of 44 asymmetrically deafened adults (Ketterer et al., 2018). In this study, subjects were effectively preselected based on the presence of HL (since they were candidates for CI), with the majority demonstrating a severe or profound degree and thus a high prevalence of tinnitus is expected. Relative to a general population in which the incidence of tinnitus is cited at 10–18% (Linnett, 2004; Al-Swiahb and Park, 2016; Vanneste et al., 2019), a much larger proportion reported tinnitus in our population at baseline (58%).

Dizziness status changed significantly between baseline and 1 year across the population, with an overall reduction in dizziness severity: the reduction was slightly greater in younger subjects than older subjects. Improvement in balance after CI has also been reported by other authors (Parietti-Winkler et al., 2015; Colin et al., 2018). Potential reasons suggested were improved spatial orientation thanks to enhanced auditory interaction with visual information, and overall improved quality of life, with follow-on effects such as resocialization leading to increased activity.

### Benefits of Cochlear Implantation for the Listening Network

Influencing the reception level of the listening network, age at implant, degree of deafness, and duration of deafness were considered. Our cohort included a wide age range of CI users (aged 18–93 years) evenly distributed across ages. Our study’s findings indicate a clear difference between younger and older subjects with the break-in age at 55 years, with 50.6% aged 55+. The older CI users reported lower baseline self-reported hearing performance from SSQ scores, but all groups demonstrated similar gains pre- to postoperatively and over time. There were no overall age-differences for HUI scores. Poorer speech understanding for deafened adults is experienced at all ages but is more pronounced in elderly subjects. We speculate that middle-age, or at least 55–65 years, is a time of transition with gradual reductions in upstream neural processes occurring. Hence, only partial restoration of hearing sensitivity with acoustic hearing aids in severe or profound hearing loss requires greater effort for middle-aged and older listeners than for younger listeners to manage verbal communication. In today’s research environment, the middle-aged group is receiving less attention, especially considering the great interest in implanting older patients underscored by the demographic shifts occurring in many western societies. Indeed, some researchers have considered this group designating them as “older” listeners (Anderson et al., 2013; Erb and Obleser, 2013). They have also been identified as a high-risk group for dementia (Sarant et al., 2020). Those 55–64 years of age may be in transition, perhaps due to the onset of synaptopathy or cognitive decline. This would significantly affect their capacity to deal with the reduced auditory sensitivity due to HL, even using hearing aids, and thus they would benefit by shifting earlier to CIs that would provide more effective, electrically transmitted auditory cues. In other words, it may be that in such a population, the listening network gradually becomes more challenged in dealing with minimal information. We note that there was no difference in SSQ scores between age groups 55–64 and 65–93, which further suggests that greater focus on a younger transition age is appropriate. Also, if treating hearing loss itself can prevent or reduce cognitive decline, as suggested in the literature (Amieva and Ouvrard, 2020), then treating it earlier would seem a logical course.

Increased problems with receptive communication for older listeners have been recognized for decades with the explanation credited to the peripheral auditory system and remediated through amplification (Dubno et al., 1984—cited by Sergeyenko et al., 2013). Shader et al. (2020) attributed the discrepancies to the aging brain being unable to process temporal information needed to understand speech. To our knowledge, our current study is the first to link the mismatch with observations from self-report data. The overriding consequence may be that younger implant users cope better with HL than older ones and can obtain higher performance postoperatively. Rehabilitation of peripheral auditory function earlier may have positive throw-on effects at the cognitive level (Felipe, 2019) because deafness-induced cortical reorganization alters the listening network, lowering its efficiency whether as a functional adaptation or a behavioral response to effortful listening.

Relative to the obvious gains obtained by remediating poor acoustic reception, the auditory system becomes increasingly more complex as it progresses through the listening network’s transmission level to the cortex (Kim et al., 2018). Presumably, in a normal-functioning transmission pathway, relatively undistorted auditory signals reach the cortex. Although CIs do not provide a perfect representation of speech input, other negative effects may degrade it further such as the reduced or interrupted capacity of the auditory nerve to relay auditory signal details. ARHL represents a complex, progressive change to the auditory system at the level of hair cell integrity and afferent synaptic interactions connecting them to the auditory nerve that may include synaptopathy (Sergeyenko et al., 2013; Tu and Friedman, 2018). It may not be surprising, then, that our findings showed a difference between the younger and older IROS participants starting in their mid-50 s when the symptoms of ARHL and synaptopathy begin to emerge. Age-group differences represented about one-third of the overall effect of CI on SSQ scores. CIs compensate for a malfunctioning transmission pathway but the implication from the SSQ scores is that older listeners cope less effectively in everyday listening conditions, which is consistent with animal studies on synaptopathy (Sergeyenko et al., 2013). Low SSQ scores may represent an indirect means of recognizing the presence of synaptopathy as a complement to currently available objective measures (Tu and Friedman, 2018; Shearer and Hansen, 2019).

There was a significant side-of-implant effect on SSQ scores: although not clinically significant (i.e., difference <2 SSQ units), right-ear CI recipients reported higher SSQ scores compared with those with left-ear CI. The size of this difference was slightly less than half the age-effect difference between <45 and 55+ years. The findings that right-ear CI users have better subjective hearing performance suggests access to a more efficient listening network where signals preferentially cross lateralize to the left hemisphere for interpretation (Scott and McGettigan, 2013). Earlier studies have not identified a significant side-of-implant effect, as in the review by Schwab et al. (2015). However, Lazard and Giraud (2017), using fMRI to studying responses of CI users, were able to identify changes at the cortical level that accompany hearing loss. Those authors propose that the adaptations that occur as a result of HL are associated with poorer CI outcomes; that is, the greater the changes, the poorer the results. They also suggested that as processing for language is generally maintained in the left hemisphere, considered the language center of the brain, the provision of sound input via a right-ear CI should provide better outcomes for speech understanding. In bilateral CI, speech perception may be optimized simply because the right-side ear will always be implanted, and we speculate that SSQ scores for quality and spatial hearing subscales may be improved over unilateral CI.

Published evidence has identified modifications in cortical activity due to lack of sensory-specific stimulation reaching the cortex, and these changes can occur rapidly (Lazard and Giraud, 2017; Han et al., 2019). Functional adaptation can be described as language networks that are constantly in flux during cognition tasks associated with speech (Plakke and Romanski, 2014). More permanent alterations may ensue whether or not the impetus for change in a mature auditory cortex results from sudden or progressive deafness. Notably, these changes are manifested as deafness-induced reallocation of cognitive resources (cross-modal reorganization, cognitive load) or the functional interaction between HL and listening (effortful listening). Age at implant, duration of HL, and experience with hearing aids have already been identified as factors affecting speech recognition in postlingually deafened adults (Kim et al., 2018). The longer the duration of deafness, the poorer the outcomes with CI (e.g., Ciscare et al., 2017; Stropahl et al., 2017; James et al., 2019). Brüggemann et al. (2017) noted a positive correlation between duration of deafness and self-reported hearing quality (using the Oldenburg Index) and Kim et al. (2018) observed a significant negative effect on word recognition scores at >10 years of deafness. Due to its slow onset and reliance on the memory of the CI user, it is difficult to validate the duration of a hearing loss (Schwab et al., 2015; Han et al., 2019). Hey et al. (2020) found no significant effect of duration of deafness on postoperative speech recognition scores once preoperative scores were entered as a covariate. We did not find an effect of duration of HL on SSQ or HUI scores (Tables s 4, 5) across intervals. Therefore, variations in the effects of duration of HL may vary due to the nature of the measurement instrument and the inclusion of covariates.

The concept of cognitive load, first mentioned by Pichora-Fuller et al. (2016), specifically relates to reallocating cognitive resources invoked in an audition. Hughes et al. (2019) define cognitive load as “mental exertion…needed to understand an auditory signal.” It is implicated in effortful listening, listening fatigue, and stressful listening, three responses describing how the brain copes with overload. Cardin (2016) point out that there is a limited capacity for mechanisms that control effort and that as processing becomes more difficult, due to hearing loss, cognitive decline may be accelerated. Faced with continued effort in listening, the main coping strategy for hearing-impaired individuals is withdrawal from difficult listening conditions, which leads to isolation, depression and is detrimental to the quality of life (Lin et al., 2011; Wayne et al., 2016; Sardone et al., 2019). Warringa et al. (2020) specifically associate coping behaviors to social loneliness manifested by adults with self-reported HL.

Although listening effort is synonymous with mental exertion, cognitive load and effortful listening are not the same; one is the mechanism and the other is the consequence. When the listening network is challenged, the brain reallocates its resources to maintain a semblance of homeostasis, compromising normal mechanisms. The changes have behavioral consequences (Luo et al., 2019). Hearing loss increases effortful listening and fatigue (Alhanbali et al., 2017; Ohlenforst et al., 2017; Warringa et al., 2020) that leads to increased cognitive load (Thomson et al., 2017) and may lead to poor test outcomes (Peelle and Wingfield, 2016; Degeest et al., 2017).

Evidence is building for an indirect impact on the recovery of cortical function as an outcome of CI, although it is not clear if the effect is to slow down the progressive nature of ARHL, to complement limited hearing (i.e., support lipreading, see Anderson et al., 2017; Lazard and Giraud, 2017) or promote plasticity (Fallon et al., 2009; Scott and McGettigan, 2013; Stahl, 2017). Our study reveals that initial gains in outcomes are maintained over 3 years, suggesting that a further deterioration in cortical processing is not indicated by reports from experienced CI users. In postlingually deafened adults, the changes occurring before CI appear to be somewhat reversible with reafferentation via electrical stimulation to restore reception of relevant auditory signals (Stropahl et al., 2017; Okamoto, 2018; Legris et al., 2018; Han et al., 2019). That is, the mature adult cortex remains relatively plastic throughout life.

### Implications for CI Candidacy

Like hearing aids, CIs directly benefit the reception level and indirectly affect all levels of the listening network. Sarant et al. (2020) have already provided solid preliminary evidence supporting the benefits of access to acoustic information in maintaining cognitive function in older subjects. The authors urge early use of auditory assistance, as do others (Roberts and Allen, 2016; Kim et al., 2018; Maharani et al., 2018). Given that the only direct prophylactic effect of hearing aids is to amplify and modify acoustic sounds that are then transformed into neural signals in a still-functioning cochlea, by extension, bypassing the cochlea to directly stimulate the auditory nerve via a CI would result in similar cortical activation. That is, if HA has the potential to slow down dementia then CIs offer the same potential. As a consequence, younger hearing-impaired adults should be offered a CI earlier rather than persevering with HAs when the acoustic input is borderline adequate. The same advice is true for hearing-impaired individuals who persevere with poor hearing and should receive hearing aids. Not waiting until changes occur due to listening effort and invoking alternative coping strategies may delay the negative changes associated with cortical decline.

We propose that implanting younger adult candidates will allow them to maintain higher levels of perceived benefit over a longer period, and especially middle-aged deafened individuals. In our study, both younger and older CI recipients report stable benefits. Transiting from HA to CI at an earlier stage when meaningful information is still arriving at the cortex and listening effort is minimal may provide younger hearing-impaired adults a more favorable opportunity to maintain their greater ability to cope with hearing loss and, thus, increase QoL years (Huinck et al., 2019). We cannot predict the consequences of ARHL or its impact on performance; however, given the intimate relationship between inadequate auditory input and cognitive decline, providing early access to the most adequate auditory input is advantageous.

### Implications for Outcome Assessment

Our study cohort demonstrated that although both younger and older CI users met candidacy requirements, which are typically based on word recognition scores <50% in quiet (or lower, depending on the country), their perceived difficulties in complex listening situations differed. The findings for our large cohort, then, bring into question whether reliance on speech discrimination scores as the gold standard for assessing candidacy and subsequent outcomes is valid. Higher postoperative scores on word and sentence testing remain a common goal but the preoperative scores may not be the definitive criterion for acceptance into a CI program, where the ultimate objective is to identify how to improve the real-life listening conditions of a hearing-disadvantaged individual. If assessments rely on speech comprehension, they may miss taking into consideration coping mechanisms needed by elderly but not younger listeners. Delaying the younger adult CI candidates may reduce their opportunity to rally active cortical regions capable of resisting negative neuroplasticity. We assert, as do Alcañiz and Solé-Auró (2018), that indications for CI candidacy need to place greater emphasis on the functional status of the hearing-impaired adult beyond the ability to recognize words in quiet or noise for understanding. In fairness, younger deafened individuals should perhaps be judged for CI candidacy based on their binaural peripheral impairment rather than their capacity to cope with severe to profound levels of hearing loss, as is currently the case in relying heavily on the importance of speech recognition scores.

If the aim is to better characterize the early onset of effortful listening, perhaps peripheral hearing measured by PTA thresholds will suffice by giving more consideration to the better ear. Applying the evaluation criterion of hearing in the better ear, Lin and associates (Lin et al., 2011) concluded that cognitive decline could be associated for each 25 dB of HL equivalent to 6.8 years of cognitive aging. Although it has been reported that PTA does not reflect synaptopathy (Tu and Friedman, 2018; Parthasarathy and Kujawa, 2018), loss of higher frequency information has been implicated in its early onset (Liberman et al., 2016; Tu and Friedman, 2018; Eggermont, 2019; Holmes and Griffiths, 2019; Profant et al., 2019). Several other language-free test paradigms have also been recommended (Fostick et al., 2013; Vannson et al., 2015; Parthasarathy and Kujawa, 2018; Holmes and Griffiths, 2019). However, conventional audiometric testing has several advantages including being cost-effective, being easier to implement than more sophisticated methods, and being highly standardized throughout the world. Routinely including higher-frequency testing (6,000–12,000 Hz) for both ears, which would complement the standard report of three- or four-frequency PTA (0.5–2 or 0.5–4 kHz, respectively), would only require the addition of adequate test headphones.

Self-report questionnaires referred to as patient-reported outcome measures (PROM), should be included routinely in assessments for CI candidacy. Degeest et al. (2017) highlight the need to pay attention to self-report HL, especially if PTA findings do not yield expected speech recognition scores as there could be a risk of delaying needed treatment and rehabilitation. If the SSQ total scores, indeed, indirectly indicate the onset of synaptopathy, then routine self-assessment of hearing difficulties should be a requirement for HA and CI candidacy, especially since such reports inherently take into consideration the contralateral ear. Further, subtle indications are more readily derived through self-report compared to standard objective measures (Hutchings and Alrubaiy, 2017). As well as the currently available PROMs, several new hearing-specific questionnaires are either being validated or are under construction as of this writing and may be worthwhile for consideration in the future (Alhanbali et al., 2017; Hughes et al., 2019).

### Implications for Rehabilitation

Post-implant rehabilitation techniques for CI recipients have focused on assisting in ways to make the best use of the new auditory signals rather than on improving the entire listening network; part of which is the act of willful or motivated hearing (Peelle, 2018). Extending the concept, then rehabilitation techniques may be well advised to include motivational therapy that enhances improvements in quality of life (Harris et al., 2016). This is not a new concept but one that is overlooked as a primary focus. Ultimately, listening improves listening (see review by Fallon et al., 2009), which emphasizes the need for rehabilitationists to motivate their CI users to persevere even if the listening experience is perceived as unsatisfactory. Vassena et al. (2019) point out that if the effort is anticipated, the resources normally utilized to instill motivation are altered. A therapy technique termed “neurorehabilitation” has been recommended applying active brain scanning during training to better understand cortical responses to therapies to help re-establish more-normal brain function in sensory-deprived individuals (Okamoto, 2018).

It has been pointed out that older users do not reach the same level of performance as their younger CI peers or a normal-hearing population (Claes et al., 2018). They further state that it is unlikely that information through a CI will ever be sufficient to allow older CI users to reach performance levels of their normal-hearing peers. As shown in this study, the range of perceived performance gains from preimplant to post-implant was similar across ages as demonstrated in both SSQ and HUI outcomes. Keeping in mind that there is clear evidence that the majority of adult deafened CI recipients show some degree of improvement on objective test comparisons of pre-post-implant scores, quality of life improvements may be the new goal of rehabilitation, which does not necessarily correlate with results on speech recognition, at least in quiet therapy settings (Capretta and Moberly, 2016). The ideal goal may be to identify whether benefits experienced in real-life situations improve for a hearing-impaired individual after cochlear implantation, not whether they reach the level of normal hearing.

### Limitations of This Study

A well-recognized limit in CI research is the absence of a control group (see comments by Sarant et al., 2020). An interesting study design has been carried out by Jayakody et al. (2018) where they compared qualified candidates who either chose to receive a CI or chose to wait for a year or more. Such an approach may offer opportunities for treatment studies. Another limitation is that there are no details of those who declined to participate in IROS but received a cochlear implant, potentially biasing the study sample.

Baseline self-reported health utility on the HUI3 scale is largely influenced by hearing in this population, and therefore other health conditions may not strongly reflect on scores and are difficult to identify as factors that may affect hearing performance, for example on the SSQ scale. Other factors may influence outcomes for CI recipients that were not captured in the IROS database, such as family support.

### Concluding Remarks

The listening network includes all functional and behavioral aspects involved in willful hearing (listening). This study focused on functional data derived from self-reported hearing performance (SSQ) and health-related quality of life (HUI) and indicated that younger CI users yield higher SSQ scores than older cohorts, that younger CI recipients demonstrating a greater degree of peripheral hearing loss, and that right-ear implantees of all ages obtain higher postoperative SSQ scores. Health-related quality of life varied similarly across ages. It is clear that younger people manage hearing loss better than older ones, and that the age gap occurs between 45 and 55 years. The study brings to light the need to focus on means and methods that mediate the effects of disabilities impacting the quality of life and healthy aging.

## Data Availability Statement

The raw data supporting the conclusions of this article will be made available by the authors, without undue reservation.

## Ethics Statement

The studies involving human participants were reviewed and approved by Health Research Ethics Committee 1, Stellenbosch University (reference N15/02/015). The patients/participants provided their written informed consent to participate in this study.

## Author Contributions

CJ and PG analyzed and interpreted data. PG was responsible for statistical analyses. CJ conceived the initial idea for the study. DM performed a literature review and initial writing. CJ and DM both extended and revised the manuscript. The remaining authors contributed the data and ensured that it was collected in a compliant manner. All authors contributed to the article and approved the submitted version.

## Conflict of Interest

CJ is an employee of Cochlear, manufacturer of Nucleus cochlear implants. PG and DM are paid consultants for Cochlear Europe. The remaining authors declare that the research was conducted in the absence of any commercial or financial relationships that could be construed as a potential conflict of interest.
